# Unveiling protection: a meta-analysis of tixagevimab-cilgavimab prophylaxis in 28,950 transplant recipients and immunocompromised patients against COVID-19

**DOI:** 10.1186/s12985-025-02814-7

**Published:** 2025-06-02

**Authors:** Mostafa Hossam El Din Moawad, Abdallah Abbas, Haneen Sabet, Mohamed Ahmed Zanaty, Abdullah Ashraf Hamad, Ayoub Rezkallah, Osama Ballut, Taha Fayad, Mona Mahmoud Elsakka, Francis Eshun, Hussien Ahmed H. Abdelgawad

**Affiliations:** 1 Faculty of Pharmacy Clinical Department, Alexandria University, Alexandria, Egypt; 2 Faculty of Medicine, Suez Canal University, Ismailia, Egypt; 3 Faculty of Medicine, Al-Azhar University, Damietta, Egypt; 4 Faculty of Medicine, South Valley University, Qena, Egypt; 5 Faculty of Medicine, Menoufia University, Shibin El-Kom, Egypt; 6 Faculty of Medicine, Algiers University, Algiers, Algeria; 7 Faculty of Medicine, Kasr Alainy, Cairo University, Cairo, Egypt; 8 Faculty of Oral and Dental Medicine, Sinai University, North Sinai, Egypt; 9 Faculty of Pharmacy, Damanhour University, Damanhour, Egypt; 10 Center for Cancer and Blood Disorders, Phoenix Children’s Hospital, Phoenix, AZ USA; 11 Department of Child Health, University of Arizona College of Medicine-Phoenix, Arizona, USA

## Abstract

**Background:**

This meta-analysis addresses the efficacy and safety of tixagevimab-cilgavimab as pre-exposure prophylaxis against COVID-19 in immunocompromised patients, particularly during the Omicron variant surge. Given the limited vaccine response in this population, alternative prophylactic strategies are critical.

**Methods:**

Following PRISMA guidelines, we comprehensively searched electronic databases, including PubMed, Scopus, Web of Science, and Embase, up to June 22, 2024. We included studies assessing tixagevimab-cilgavimab's impact on SARS-CoV-2 infection rates, hospitalization, ICU admissions, and/or mortality among immunocompromised patients. Data synthesis and analysis were conducted using RevMan and Open-Meta Analyst software.

**Results:**

Analyzing data from 36 studies involving 28,950 patients, tixagevimab-cilgavimab significantly reduced SARS-CoV-2 infection rates by 4.37%, hospitalization by 0.8%, and mortality by 0.5%. Compared to no prophylaxis, the drug combination showed a notable reduction in SARS-CoV-2 infection (OR = 0.33, 95% CI: 0.22–0.50), hospitalization (OR = 0.24, 95% CI: 0.15–0.39), and mortality (OR = 0.33, 95% CI: 0.16–0.66), exhibiting a favorable safety and efficacy profile. During the Omicron surge, tixagevimab-cilgavimab consistently reduced infection risk (OR = 0.32, 95% CI: 0.17–0.58).

**Conclusion:**

Tixagevimab-cilgavimab offers a significant protective effect against COVID-19, including Omicron variants, in immunocompromised patients, underscoring its role as an effective pre-exposure prophylaxis. Future studies should further explore its efficacy across different SARS-CoV-2 variants and potential synergies with vaccination efforts.

**Supplementary Information:**

The online version contains supplementary material available at 10.1186/s12985-025-02814-7.

## Introduction

Quite recently, considerable attention has been given to the methods used to decrease coronavirus disease 2019 (COVID-19)-related complications and mortality in specific groups, such as immunocompromised patients. COVID-19 vaccine remains the cornerstone to prevent severe illness, hospitalization, and death from SARS-CoV-2 infection. Certain populations showed a reduced response to COVID-19 vaccines compared with healthy individuals, among whom immunocompromised patients [1, 2], primarily cancer, stem cell transplant (SCT), and solid organ transplant patients [3].

The United States Food and Drug Administration (FDA) authorized tixagevimab plus cilgavimab (Evusheld™) for pre-exposure prophylaxis (PrEP) for the prevention of COVID-19 caused by the SARS-CoV-2 virus under emergency use authorization [4]. It is intended for adults and adolescents with moderate to severe immune compromise due to a medical condition or immunosuppressive treatments, making them unlikely to respond adequately to COVID-19 vaccination, or those who cannot receive a COVID-19 vaccine.

Tixagevimab-cilgavimab is a neutralizing monoclonal antibody regimen (mAbs) directed against different epitopes of the receptor-binding domain (RBD) of the SARS-CoV-2 spike protein, providing passive immunization in immunocompromised patients [5]. The dose and administration of tixagevimab-cilgavimab are as follows: 300 mg tixagevimab and 300 mg cilgavimab administered as two consecutive intramuscular injections at different sites in two different muscles [5]. The phase III PROVENT study demonstrated a breakthrough infection rate of 0.5% in unvaccinated adults at an increased risk of inadequate response or exposure to COVID-19 [6]. Several resistant variants of the virus have appeared, such as BA.1 and BA.2 SARS-CoV-2 Omicron sublineages presented different degrees of immunization after treatment with monoclonal antibodies [7].

There has been a conflict over whether to use tixagevimab-cilgavmab as PrEP in immunocompromised patients and whether it is effective after the appearance of new variants, such as the omicron variants as supposed by the World Health Organization (WHO) [8].

Thus, we performed this meta-analysis to elucidate and assess the risk of SARS-CoV-2 infection and its subsequent outcomes among transplant recipients and other immunocompromised patients receiving tixagevimab-cilgavimab as a PrEP, in addition to evaluating its safety profile and potential adverse effects.

## Methods

We followed the Preferred Reporting Items for Systematic Reviews and Meta-analyses (PRISMA) statement guidelines when reporting this systematic review and meta-analysis [7]. All steps were done in strict accordance with the Cochrane Handbook of Systematic Reviews and Meta-analysis of Interventions, version 6.4 [9].

### Eligibility criteria

Our systematic review and meta-analysis incorporated studies that satisfied specific criteria, including studies involving immunocompromised patients, regardless of the underlying cause or COVID-19 vaccination status. We also considered studies where the experimental group received the tixagevimab-cilgavimab combination, either with or without a comparator. Our inclusion criteria included various study designs, including observational studies (cohort, cross-sectional, and case–control) and clinical trials. Conversely, we excluded studies with unreliable data for extraction and analysis, those reported solely as theses, investigations of the tixagevimab-cilgavimab combination in immunocompetent individuals, case reports, case series, reviews (both narrative and systematic), and previously conducted meta-analyses.

### Search strategies and selection criteria

We extensively searched four electronic databases (PubMed, Scopus, Web of Science, and Embase) from their inception until June 22, 2024. The articles were retrieved using title and abstract in PubMed, Embase, and Web of Science in addition to title, abstract, and keywords in Scopus. The resulting articles were then exported using Research Information Systems (RIS) files. The search strategy employed the following keywords: COVID-19, immunocompromised, and tixagevimab-cilgavimab. The full search strategy is illustrated in Supplementary Table 1. Further, the references of the included studies were manually searched for any potentially eligible studies.

### Selection process

All search results were imported into the Rayyan software [9], where the duplicates were identified and removed, and the remaining references underwent screening. Selection of eligible studies was performed by initial screening of the titles and abstracts for relevance, followed by full-text article screening using the pre-specified inclusion and exclusion criteria. Any discrepancies during the screening process were resolved by consulting a senior author.

### Data collection process and data items

Data were systematically extracted and compiled using Google Sheets onto a standardized data extraction sheet**.** The extracted data encompassed three key categories:

- (a) A summary of the included studies (study design, sample size, dose, medication period, follow-up period, the aim of the study, and main findings).
- (b) Baseline characteristics of the study populations, including age, gender distribution, and causes of immunosuppression.
- (c) Outcome measures covering rates of post-tixagevimab-cilgavimab COVID-19 infection, hospitalization, ICU admission, and deaths.

### Quality assessment

Quality assessment for observational studies was conducted using the National Institute of Health (NIH) tool [10]. Studies with a score of one to four were considered of poor quality, five to eight of fair quality, and more than eight of high quality. The risk of bias in randomized controlled trials was assessed using the Cochrane risk of bias tool. (Rob2) [11].

### Effect measures

In the present meta-analysis, we considered the following outcome measures among patients who received the tixagevimab-cilgavimab combination: overall SARS-Cov-2 infection rate, overall hospitalization rate, COVID-19-specific hospitalization, ICU admission, overall mortality rate, COVID-19-specific mortality, and adverse events associated with tixagevimab-cilgavimab. When controlled clinical trials were included in the analysis, direct comparisons between tixagevimab-cilgavimab and placebo were conducted regarding the risks of SARS-Cov-2 infection, hospitalization, and mortality, as applicable.

### Statistical analysis

The primary endpoints of this meta-analysis were to evaluate the risks of SARS-Cov-2 infection, hospitalization, ICU admission, and mortality associated with the use of the tixagevimab-cilgavimab combination. The secondary endpoint was the incidence of adverse events and serious adverse events associated with administering the tixagevimab-cilgavimab.

Pooled analyses were performed to calculate the odds ratios (OR) for each outcome. In the comparative analysis, the OR quantifies the relative odds of experiencing the event of interest in the tixagevimab-cilgavimab group compared to the placebo group.

The Review Manager version 5.4 [9] software was employed for these analyses. The ORs were determined using the Mantel–Haenszel method, and 95% confidence intervals (CIs) were calculated around the odds ratios to gauge the level of precision in the results. To include data from single-arm trials and observational studies involving patients who utilized the tixagevimab-cilgavimab and to calculate the overall mortality and infection rates, the Open-Meta Analyst software 2021 was employed [12].

A random-effects model was employed for the pooled analyses due to the anticipated variability in study populations, interventions, and study designs. Random-effect models were implemented at a confidence level of 95%, and a *p*-value < 0.05 was considered statistically significant.

Heterogeneity among the included studies was assessed using I^2^ and was deemed statistically significant if it was less than or equal to 0.05. Sensitivity analyses were conducted to assess the robustness of the results by excluding studies with potential sources of bias or studies with small sample sizes using Open-Meta Analyst software. Publication bias was assessed using funnel plots constructed by Review Manager software.

## Results

### Search and screening

**Fig. 1:**
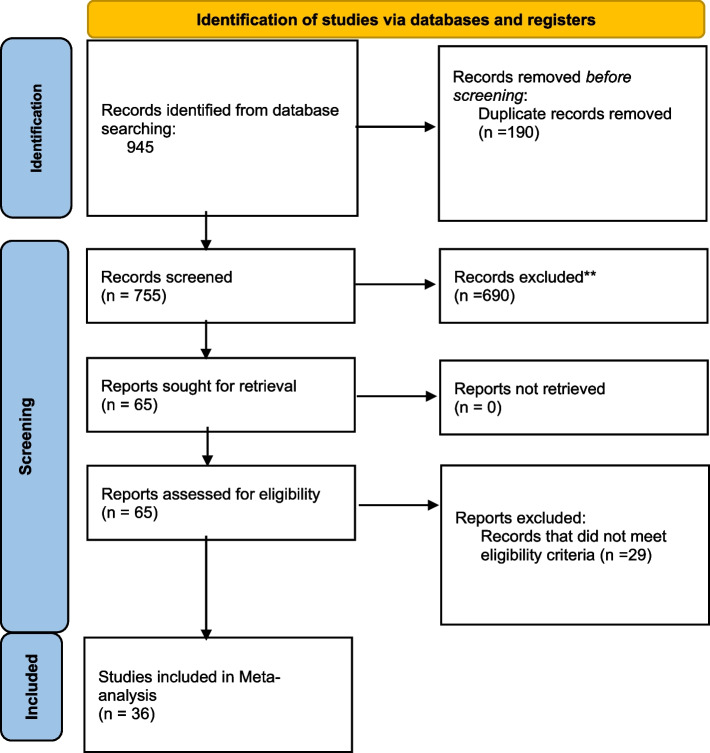
PRISMA flow diagram of search and screening processes

The database resources engaged for our inquiry yielded a comprehensive collection of 945 research articles for thorough examination. Following duplicate removal, 755 distinct articles were retained for initial assessment. Subsequent to a meticulous evaluation of titles and abstracts, 690 articles were deemed ineligible for further consideration. After a rigorous screening process, 36 studies [6, 13–47] fulfilled our eligibility criteria and were deemed suitable for inclusion within the systematic review framework and subsequent meta-analysis, as shown in Fig. 1.

### Risk of bias assessment

Out of the 36 included cohort studies, 24 were of fair quality, while nine were of poor quality as per the NIH tool, as shown in Supplementary Table 2. Regarding the RCTs, the risk of bias assessment using the Rob2 tool showed that two had some concerns, and one had a low risk of bias, as shown in Supplementary Fig. 1.

### Baseline characteristics

Among the included studies, 33 were observational cohort studies, and three were RCTs. Twelve studies used a combination of 150 mg of tixagevimab and 150 mg of cilgavimab, while seven studies used 300 mg of each, two studies used 600 mg of each, and 11 studies used one of both doses in different patients. The follow-up period ranged from one to six months. The included patients were immunosuppressed due to a variety of diseases, such as hematological malignancies and autoimmune diseases. Others were on immunosuppressive therapies or were transplant recipients. The baseline characteristics and the summary of the included studies are shown in Table 1 and Supplementary Table 3.

**Table 1:** Baseline characteristics of the included studies

Study ID	Study design	Sample size		Age, mean (SD)		Gender (m/f)		Cause of immunosuppression	Dose	Follow-up, months
		Drug	Control	Drug	control	Drug	control			
Al-Obaidi 2023 [35]	Cohort	463 patients	-	67 (3.40)	-	238/225	-	Hematologic malignancies, transplant, autoimmune diseases, advanced HIV, and solid tumors on chemotherapy	300\300 mg	1
Calabrese 2022 [21]	Cohort	12	-	64^*^	-	4/8	-	Potent immunosuppressives	300/300 mg or 150 mg/150 mg	-
Jakimovski 2023 [36]	Cohort	31	126	55.1 (10.3)	51.2 (12.4)	12/19	40/86	Potent immunosuppressives	150\150 mg changed to 300\300 mg	6
Jondreville 2022 [30]	Cohort	161	-	52.58(9.93)	-	-	-	Transplant recipients	150\150 mg	3.5
Karaba 2023 [26]	Cohort	36		64.4(1.50)		-		Transplant recipients	300 mg/300 mg or 150 mg/150 mg	3
Kertes 2023 [15]	Cohort	825	4299	-	-	512/313	2291/2008	Immune-compromised condition/treatment hypogammaglobulinemia, chronic lymphocytic leukemia, anti-CD20 Rx in last 6 months, bone marrow transplant, chimeric antigen receptor T-cell therapy, solid-organ transplant, lymphoma, and multiple myeloma	150 mg/150 mg	1.77*
Montgomery 2022 [14]	RCT	452	451	46·3 (15·4)	45.9 (15.0)	213/239	235/216	Chronic diseases and immunocompromised patients	300\300 mg	2.8*
Nguyen 2022 [29]	Cohort	1112	-	-	-	-	-	Kidney, heart, lung, or liver transplant recipients; hematologic malignancies, rituximab for autoimmune diseases, other immunosuppressive treatments, and primary immune deficiency	150/150 mg	2.07
Ocon 2022 [32]	Cohort	203	-	72 (10)	-	109/94	-	Hematological malignancy	300/300 mg or 150 mg/150 mg	6
Ordaya 2022 [18]	Cohort	8	-	53.63(22)	-	2/6	-	Transplant recipients	150/150 mg	-
Ordaya 2023 [24]	Cohort	25	-	61(17.30)	-	12/13	-	Solid-organ transplant, hematopoietic stem-cell transplant, hematologic malignancy, and autoimmune disorder	300/300 mg	-
Totschnig 2022 [34]	Cohort	116	59.6 (15.1)	-	-	53/63	-	Hematologic malignancy, autoimmune disease, multiple sclerosis, immunodeficiency, and organ transplantation	150/150 mg	3.13
Ocon 2023 [33]	Cohort	43		59 (15)	-	13/30	-	Rheumatoid arthritis, vasculitis, immune-mediated myositis, Sjögren disease, systemic lupus erythematosus, systemic oral corticosteroids, methotrexate, azathioprine, leflunomide, sulfasalazine, mycophenolate, and avacopan	300/300 mg or 150 mg/150 mg	3.33
Connolly 2023 [25]	Cohort	11	-	-	-	2/9	-	All patients were treated with at least one immunosuppressant, most commonly mycophenolate	300/300 mg or 150 mg/150 mg	-
Al Jurdi 2022 [38]	Cohort	222	222	64.25(3.06)	63 (2.89)	136/86	130/92	Solid organ transplant	300/300 mg or 150 mg/150 mg	2.9
Alejo 2023 [28]	Cohort	392	-	-	-	164/228	-	Solid organ transplant	150/150 mg	3
Benotmane 2022 [17]	Cohort	39	-	59.1(14.53)	-	23/16	-	Kidney transplant recipients	150/150 mg	-
Bertrand 2022 [22]	Cohort	145	98	-	-	-	-	Kidney transplant recipient	150/150 mg	2.67
Chen 2022 [31]	Cohort	1295	-	58.78(12.27)	-	746/549	-	Solid organ transplant recipients, bone marrow transplants, hematologic malignancies, and other qualifying conditions (including active chemotherapy, advanced HIV/AIDS, significant immunosuppression for an autoimmune disorder, etc.)	150/150 mg	
Cochran 2023 [20]	Cohort	205	-	-	-	-	-	Solid organ transplant recipients	300/300 mg or 150 mg/150 mg	3.1*
Davis 2023 [6]	Cohort	251	-	60.25(13)	-	F149/102	-	B-cell malignancies	300/300 mg or 150 mg/150 mg	-
Gottlieb 2023 [23]	Cohort	419	1019	59.25(2.19)	56(2.78)	221/198	553/467	Lung transplant	300/300 mg or 150 mg/150 mg	6.63
Kaminski 2022 [19]	Cohort	333	97	60 (14.4)	58.3 (14.3)	204/129	63/34	Kidney transplantation	150/150 mg	3.87
Levin 2022 [13]	RCT	3460	1737	53.6 (15.0)	53.3 (14.9)	1865/1595	935/802	Obesity, hypertension, smoking, diabetes, asthma, cancer, cardiovascular disease, chronic kidney disease, chronic obstructive pulmonary disease, chronic liver disease, immunosuppressive treatment, sickle cell disease, and immunosuppressive disease	300/300 mg	6*
Levin 2023 [16]	RCT	749	372	46.6 (15.7)	46.0 (16.2)	376/373	191/181	Obesity, hypertension, smoking, diabetes, asthma, cancer, cardiovascular disease, chronic kidney disease, chronic obstructive pulmonary disease, chronic liver disease, immunosuppressive treatment, sickle cell disease, and immunosuppressive disease	300/300 mg	1.82
Marchesi 2023 [27]	Cohort	45	45	67.25(1.59)	71(3.18)	26/19	26/19	Hematological malignancy	-	3.44
Young-Xu 2022 [37]	Cohort	1,733	6354	68.1 (11.5)	67.4 (11.0)	1579/154	5796/558	Immunocompromised conditions and immunosuppressants use	300/300 mg or 150 mg/150 mg	4
Lombardi 2023 [45]	Cohort	19	89	-	-	9/19	52/89	- history of any connective tissue disease, autoimmune disease, or primary immunodeficiency-history of an active solid or hematologic tumor- neutropenia due to hematological cancer- (HIV) infection or acquired immunodeficiency syndrome (AIDS)- history of splenectomy, solid organ transplantation (SOT), and/or hematopoietic stem cell transplantation (HSCT)-ongoing treatment with steroids (for at least four weeks), chemotherapy, and/or immunosuppressive agents	-	14 day
Benotmane 2023 [47]	Retrospective cohort	36	98	-	-	22/36	69/98	Kidney transplantation	600 mg	-
Trepl 2024 [41]	Retrospective cohort	40	26	Total: 54.7 (15.2)		Total: 46/66		hematopoietic stem cell transplantation (HSCT)	300/300 mg	267 days*
Angelico 2023 [40]	Cohort	35	25	Total: 59.52 (12.91)		Total: 34/60		Kidney and liver transplantation	-	12
Sindu 2023 [44]	Retrospective cohort	24	65	-	-	14/24	35/65	Lung transplantation	300/300 mg	-
Demolder 2024 [39]	Retrospective Cohort	264	-	63.33 (9.69)	-	128/136	-	Lung transplantation, tacrolimus-based immunosuppressive regimen	150 mg/150 mg	6
Fraczkiewicz 2024 [42]	Cohort	78	-	15.3 (2.2)	-	53/25	-	hemato-oncological, hematopoietic stem cell transplantation, chimeric antigen receptor T-cell	600 mg/600 mg	8.16*
AA Roppelt 2023 [43]	Cohort	48	-	44.33 (44.33)	-	21/27	-	Primary immunodeficiencies (PIDs)	150 mg/150 mg	5.8*
Lichvar 2023 [46]	Cohort	481	-	58.8	-	312/169	-	Lung transplant and solid organ transplant (SOTR)	-	3.6*

*ID* Identification, *RCT* Randomized Controlled Trial, *m/f* Male/Female, *SD*: Standard Deviation, -: Data not available, *HIV* Human Immunodeficiency Virus, *HSCT* Hematopoietic Stem Cell Transplantation, *SOTR* Solid Organ Transplant Recipients, *PIDs* Primary Immunodeficiencies, *B-cell* Type of white blood cell important for immune response, *Rx* Treatment, *mg* Milligram

*data expressed as median

### Risk of SARS-CoV-2 infection

**Fig. 2:**
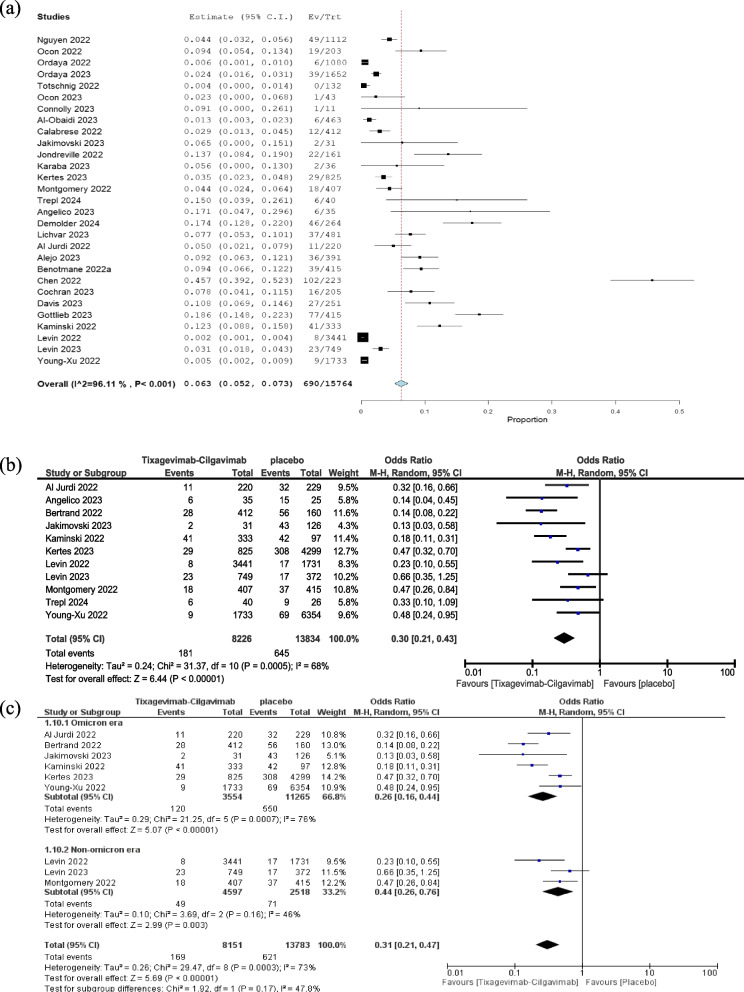
Overall infection rate in patients taking tixagevimab-cilgavimab combination (**a**), comparison between patients taking tixagevimab-cilgavimab combination and control regarding infection rate (**b**), and its subgroup analysis according to Omicron era (**c**)

The analysis of 29 studies involving a cohort of 15,764 immunocompromised patients revealed a 6.3% incidence (Fig. 2A) of SARS-CoV-2 infection among those who received tixagevimab-cilgavimab.

In a meta-analysis of data from 11 studies, it was found that using tixagevimab-cilgavimab PrER significantly lowered the risk of SARS-CoV-2 infection when compared to not using any prophylaxis (OR = 0.3, 95% CI: 0.21–0.43, *P* < 0.00001, Fig. 2B). Pooled studies were heterogeneous (I^2^ = 68% and *P* = 0.0005). To address this heterogeneity, we conducted a leave-one-out analysis, showing that Bertrand et al., 2022, and Levin et al., 2023, were the sources of heterogeneity [16, 22] (Supplementary Fig. 2). Using data restricted to the omicron era of the pandemic, tixagevimab-cilgavimab did not lose its effectiveness in reducing the risk of SARS-CoV-2 infection when compared to no prophylaxis (OR = 0.26, 95% CI: 0.16, 0.44, *P* < 0.00001, Fig. 2C).

### Risk of hospitalization

**Fig. 3:**
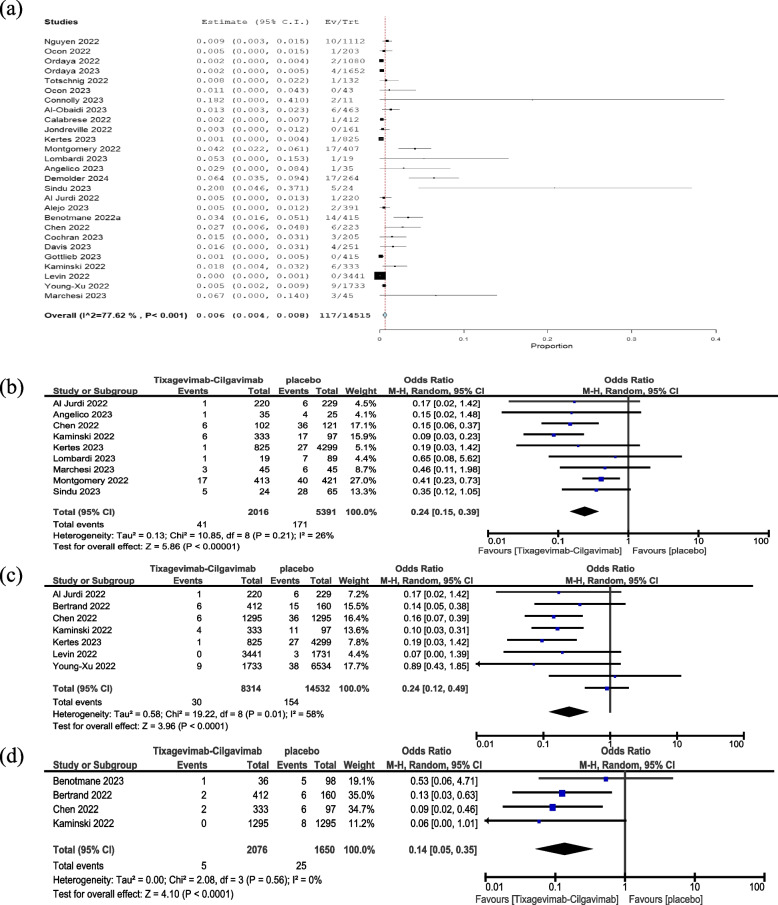
Overall hospitalization rate in patients taking tixagevimab-cilgavimab combination (**a**), comparison between tixagevimab-cilgavimab and control regarding, (**b**) overall hospitalization, (**c**) COVID-19 specific hospitalization, and (**d**) ICU admission

The analysis of 27 studies involving a cohort of 14,515 immunocompromised patients revealed a 0.6% incidence of hospitalization among those who received tixagevimab-cilgavimab (Fig. 3A).

In a meta-analysis of data from nine studies, using tixagevimab-cilgavimab PrER was associated with a lower risk of hospitalization (OR = 0.24, 95% CI: 0.15–0.39, *P* < 0.00001) (Fig. 3B). Pooled studies were homogenous (I^2^ = 26% and *P* = 0.21). Moreover, COVID-19-specific hospitalization rates were significantly lower in the tixagevimab-cilgavimab group (OR = 0.24, 95% CI: 0.12–0.49, *P* < 0.00001, Fig. 3C). Pooled studies were heterogeneous (I^2^ = 58% and *P* = 0.01). To address this heterogeneity, we conducted a leave-one-out analysis, and it showed that Young-Xu et al. [37] was the source of heterogeneity. (Supplementary Fig. 3).

### Risk of ICU admission

In a meta-analysis of data from four studies, it was found that using tixagevimab-cilgavimab PrER significantly lowered ICU admission rates when compared to not using any prophylaxis (OR = 0.14, 95% CI: 0.05–0.35, *P* < 0.0001, Fig. 3D). Pooled studies were homogenous (I^2^ = 0% and *P* = 0.56).

### Risk of Mortality

**Fig. 4:**
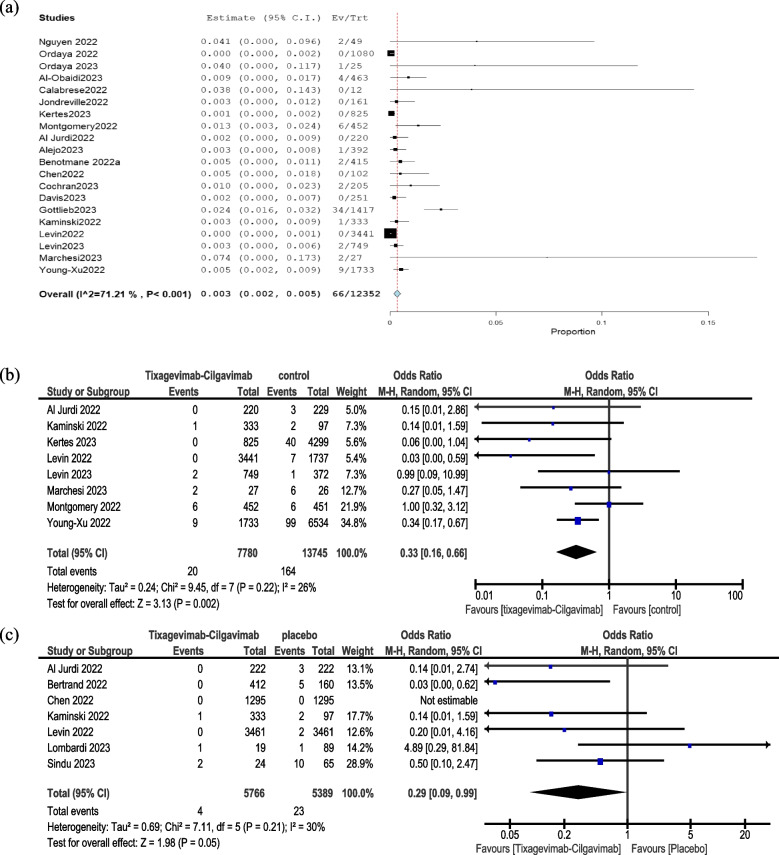
Overall mortality in patients taking tixagevimab-cilgavimab combination (**a**), comparison between tixagevimab-cilgavimab combination and control regarding overall mortality (**b**), and COVID-19 specific mortality (**c**)

The analysis of 20 studies involving a cohort of 12,352 immunocompromised patients revealed a 66 (0.3%) incidence (Fig. 4A) of mortality among those who received tixagevimab-cilgavimab.

In a meta-analysis of data from eight studies, it was found that using tixagevimab-cilgavimab PrER significantly lowered the mortality rate when compared to not using any prophylaxis (OR = 0.33, 95% CI: 0.16–0.66, *P* = 0.002, Fig. 4B). Pooled studies were homogenous (I^2^ = 26% and *P* = 0.22).

In a meta-analysis of data from seven studies, it was found that using tixagevimab-cilgavimab PrER significantly lowered the COVID-19-specific mortality rate when compared to not using any prophylaxis (OR = 0.29, 95% CI: 0.09–0.99, *P* = 0.05, Fig. 4C). Pooled studies were homogenous (I^2^ = 30% and *P* = 0.21).

### Safety analysis

Injection site reactions were the most commonly reported adverse events in patients who received tixagevimab-cilgavimab. The pooled analysis of three studies revealed no significant difference between the tixagevimab-cilgavimab group and the control groups in terms of the incidence of adverse events (OR = 0.81, 95% CI: 0.58–1.12, *P* = 0.21, Supplementary Fig. 4 A). Moreover, three studies reported the incidence of severe adverse events (SAEs). The overall incidence of SAEs was comparable between tixagevimab-cilgavimab and control groups (OR = 0.90, 95% CI: 0.59–1.36, *P* = 0.61, Supplementary Fig. 4B).

### Publication bias

We conducted publication bias for the the risk of “SARS-CoV-2 infection” outcome using a funnel plot which showed minimal risk of publication bias. (Supplementary Fig. 5).

## Discussion

The Food and Drug Administration (FDA) recently issued a communication indicating that the monoclonal antibody combination of tixagevimab and cilgavimab is no longer authorized for emergency use in the United States [8]. This directive was disseminated amidst concerns regarding the potential of the Omicron variant of SARS-CoV-2 to resist neutralization by tixagevimab and cilgavimab; therefore, it may not confer protection against this subvariant. Conversely, our investigation showed that tixagevimab-cilgavimab did not lose its effectiveness in reducing the risk of SARS-CoV-2 infection in immunocompromised patients during the Omicron era of the pandemic.

The World Health Organization's Director-General proclaimed the demotion of COVID-19 from its status as a global health emergency [48]. Although this announcement marks a significant transition in the international management of the pandemic, it is accompanied by a caveat emphasizing that the disease continues to pose a considerable threat to public health across the globe. This assertion is particularly pertinent for demographic segments characterized by immunocompromised conditions, such as individuals who have undergone stem cell transplantation or those diagnosed with malignancies.

Despite intensified efforts to vaccinate these populations, there exists a notable proportion—approximately 20%—of transplant recipients who do not develop an adequate humoral response to the vaccination [49]. A systematic review and meta-analysis involving 11,713 solid organ transplant recipients found that the seroconversion rates improved with the number of mRNA vaccine doses: 10.4% after one dose, 44.9% after two doses, and 63.1% after three doses. However, even after three doses, a notable proportion of recipients still exhibited inadequate humoral responses, with some studies reporting that up to 50% of recipients had minimal or no antibody response [50]. In this context, the utilization of tixagevimab-cilgavimab as a pre-exposure prophylactic agent against SARS-CoV-2 infection has been endorsed for this vulnerable cohort, grounded on the empirical findings from the PROVENT study [13]. Also, sotrovimab was tested in solid organ transplant recipients and showed efficacy and improved COVID-19-related outcomes [51].

Our meta-analysis revealed that the use of tixagevimab-cilgavimab in immunocompromised patients significantly reduces the likelihood of COVID-19 infection compared to a placebo. This conclusion was drawn from a meta-analysis of 11 studies, encompassing 8,226 immunocompromised individuals receiving tixagevimab-cilgavimab and 13,834 placebo recipients. Furthermore, we examined the rate of COVID-19 infection across 29 studies involving 15,764 participants treated with tixagevimab-cilgavimab, discovering an infection rate of merely 6.3%. Our findings confirm the tixagevimab-cilgavimab combination's effectiveness in preventing COVID-19 infection during both the Omicron and pre-Omicron periods. Moreover, our analysis further demonstrated that tixagevimab-cilgavimab usage is linked to reduced mortality rates compared to placebo. This significant effect highlights the drug combination's efficacy in decreasing both overall and COVID-19-specific mortality rates, showcasing its value in managing the disease's impact on high-risk populations. Additionally, tixagevimab-cilgavimab significantly reduces the risk of hospitalization and ICU admission for COVID-19 compared to placebo, indicating the drug combination's effectiveness in preventing severe disease outcomes. The safety of the drug combination is a major concern since the target population has a diminished immunity.

Our results found that the tixagevimab-cilgavimab combination did not significantly increase the risk of adverse events or severe adverse events compared to placebo in immunocompromised patients. Both groups reported similar incidences of mild to moderate adverse effects, including injection site pain. Notably, the drug combination group experienced fewer COVID-19-related deaths and lower all-cause mortality rates. These safety findings are consistent with those from the phase 3 PROVENT and STORM CHASER studies, affirming the drug's safety profile in the target population [13, 16].

The meta-analyses by Soeroto et al. [52] and Suribhatla et al. [53], while aligning with our findings on the efficacy and safety of tixagevimab-cilgavimab for COVID-19 prophylaxis in immunocompromised patients, were based on relatively small numbers of studies. Their findings corroborate our results, demonstrating a decrease in COVID-19 infection rates, hospitalizations, and mortality, along with reduced severity of symptoms. Both studies highlight the drug combination's clinical efficacy in reducing the risk of COVID-19 infection and its associated outcomes, aligning with our observations on its effectiveness and safety profile. Khorramnia et al. [54] conducted a meta-analysis to investigate tixagevimab-cilgavimab combination in solid organ transplant recipients and showed the efficacy of the combination in lowering the infection rate, hospitalization rate, and intensive care unit admission, as in our study, but no effect was observed on mortality, which contraindicates our findings. This meta-analysis was limited by small sample size in some outcomes, and the evidence was of a low to moderate level.

Although the PROVENT and STORM CHASER trials [13, 16] didn’t investigate the efficacy of tixagevimab-cilgavimab in the prevention of Omicron subvariants, studies conducted in vitro have demonstrated that the drug combination maintains its ability to neutralize the BA.1, BA.1.1, BA.2, BA.2.12.1, BA.3, BA.4, and BA.5 Omicron subvariants, with a potency falling within the IC50 geometric mean concentration range of 4.0–806.0 ng/mL [55–59]. Since the anti-SARS-CoV-2 neutralizing antibody titer that manifests in sera after delivery is larger than that of convalescent serum, it is anticipated that AZD7442 will be clinically effective against these Omicron subvariants [60]. The BA.2 and BA.4/5 Omicron subvariants remain susceptible to tixagevimab-cilgavimab, with IC50 values indicating effective neutralization at low concentrations [57, 58, 61].

For optimal pre-exposure COVID-19 prophylaxis, a revised dosage of 600 mg AZD7442, split equally between tixagevimab and cilgavimab, is recommended every six months, aiming to provide robust protection against these and potentially emerging variants [4]. In-vitro data indicate that tixagevimab-cilgavimab's effectiveness is reduced against Omicron subvariants BA.4/5, BQ.1.1, and XBB.1.5 [58, 62]. However, serum levels of these antibodies are much higher than needed for significant efficacy against the ancestral virus, suggesting that clinical efficacy may be retained despite reduced in-vitro activity against these variants. Solera and colleagues [63] conducted a study on organ transplant recipients and found that tixagevimab-cilgavimab effectively neutralized the BA.4/5 Omicron subvariant in most cases, despite no significant impact on BQ.1.1 and XBB.1.5 variants. This highlights its potential for specific Omicron subvariants but limited utility for others in severe COVID-19 risk patients. In a cohort of 205 solid organ transplant recipients receiving tixagevimab-cilgavimab, low COVID-19 incidence, hospitalization, and mortality rates were reported among solid organ transplant recipients who received tixagevimab-cilgavimab during the Omicron surge [20].

Additionally, Davis et al. initially demonstrated the efficacy of tixagevimab-cilgavimab in preventing breakthrough COVID-19 infections among patients with hematological malignancies, particularly against the prevalent Omicron BA.5 variant [6]. Totschnig et al.'s study differentiated the antibody response between sotrovimab and tixagevimab-cilgavimab, noting sotrovimab's initial higher antibody levels but highlighting tixagevimab-cilgavimab's advantage in longer-term immunity due to its extended half-life and different administration route, suggesting a sustained protective effect in immunocompromised patients [34].

Furthermore, participants in the STORM CHASER study had not been previously vaccinated against COVID-19. Nonetheless, evidence indicates that tixagevimab-cilgavimab does not compromise the immunogenicity of COVID-19 vaccines [64]. In fact, preliminary findings suggest that tixagevimab-cilgavimab might enhance the protective response against SARS-CoV-2, especially in immunocompromised individuals who have completed their vaccination regimen, potentially bolstering their defenses against the virus [65, 66].

The recently published meta-analysis by Glhoom et al. [67] investigated the potential increase in tixagevimab-cilgavimab dose from 300 to 600 mg with a booster dose after six months, as recommended by the WHO with the new variant, and showed that there was no significant difference in efficacy between the two doses for preventing COVID-19.

Our study represents the most exhaustive analysis to date on the efficacy and safety of the tixagevimab-cilgavimab combination as a prophylactic measure against COVID-19, including its Omicron variants, in immunocompromised patients. Incorporating data from all available clinical trials and observational studies, our research covers approximately 28,950 patients treated with tixagevimab-cilgavimab. We thoroughly examined various outcomes related to the risk and consequences of infection, providing a comprehensive understanding of the drug combination's impact. Despite this, our study faced several limitations, including the combination of observational studies with clinical trials in the same analysis. The influence of prior COVID-19 infections on current antibody levels wasn't considered, nor was the vaccination status of participants in some studies. Additionally, variations in the causes of immunosuppression among participants could affect infection rates and responses. Future research should address these confounding factors by considering the diversity of diseases and immunosuppressive treatments to provide a more nuanced understanding of the drug combination's efficacy and safety.

## Conclusion

Our comprehensive meta-analysis underscores the efficacy and safety of tixagevimab-cilgavimab for COVID-19 prophylaxis in immunocompromised patients, notably transplant recipients. We've integrated data from various studies, revealing significant reductions in infection, hospitalization, ICU admissions, and mortality rates, alongside a favorable safety profile.

Incorporating the latest insights on the Omicron variant's emergence and its sublineages, our meta-analysis robustly affirms the prophylactic potential of tixagevimab-cilgavimab in immunocompromised populations, including those undergoing organ transplantation. Despite the evolving landscape of SARS-CoV-2 mutations, our findings highlight a significant protective effect against COVID-19 infection, severe disease progression, and related mortality, with a well-tolerated safety profile. Acknowledging the dynamic nature of viral evolution, we advocate for ongoing research to adapt prophylactic strategies to emerging SARS-CoV-2 variants and to explore the integration with COVID-19 vaccination efforts for enhanced patient outcomes.

## Supplementary Information

Supplementary Material 1.

## Data Availability

This systematic review and meta-analysis relied on publicly available data from previously published studies. The original research contributions utilized in this study can be accessed within the main article and supplementary materials.
