# Supplementary material for: Unveiling protection: a meta-analysis of tixagevimab-cilgavimab prophylaxis in 28,950 transplant recipients and immunocompromised patients against COVID-19
Source: Virol J. 2025 Jun 2;22:178. doi: 10.1186/s12985-025-02814-7 (PMC12131589; doi:10.1186/s12985-025-02814-7)
Supplement: Supplementary file 1 — Supplementary Material 1. [file 12985_2025_2814_MOESM1_ESM.docx]

**Supplementary Table 1: Search strategy of the present study**

| **Search strategy** | (“coronavirus” OR “corona virus” OR “covid 2019” OR “SARS2” OR “SARS-CoV-2” OR “2019ncov” OR “sars cov2” OR “cov22” OR “covid-19” OR “covid19” OR “covid” OR “coronaviridae”) AND (“tixagevimab” OR “cilgavimab” OR “tixagevimab cilgavimab” OR “tixagevimab-cilgavimab” OR “AZD7442”) AND (“cancer” OR “malignancy” OR “malign*” OR “immunocompromise” OR “immunocompromised” OR “immunodeficiency” OR “immunodeficient” OR “immunodef*” OR “immunocompr*” OR “chemotherapy” OR “chemo*” OR “immunosuppressed” OR “immunosuppression” OR “immunosupp*” OR “rheumatology” OR “rheumatic” OR “rheum*” OR “autoimmune” OR “autoimmunity” OR “immunosuppressive” OR “steroid*” OR “corticosteroid*” OR “antineoplastic*” OR “chemotherap*”). |
| --- | --- |

**Supplementary Table 2: Risk of bias assessment for observational cohort studies using the NIH tool**

| **Study ID** | **1** | **2** | **3** | **4** | **5** | **6** | **7** | **8** | **9** | **10** | **11** | **12** | **13** | **14** | **overall** |
| --- | --- | --- | --- | --- | --- | --- | --- | --- | --- | --- | --- | --- | --- | --- | --- |
| Al-Obaidi 2023 | Y | Y | NA | Y | N | N R | Y | NR | NA | NA | NR | NR | NA | NR | Poor |
| Calabrese 2022 | Y | Y | NA | Y | N | NR | NR | NR | NA | NA | NR | NR | NA | NR | Poor |
| Jakimovski 2023 | Y | Y | NR | Y | N | NR | Y | NR | NA | NA | Y | NR | NR | NR | Fair |
| Jondreville 2022 | Y | Y | NA | Y | N | Y | Y | NR | NA | NA | NR | NR | NR | NR | Fair |
| Karaba 2023 | Y | Y | NR | Y | N | Y | Y | N | NA | NA | Y | NR | NR | NR | Fair |
| Kertes 2023 | Y | Y | NA | Y | N | Y | NR | N | NA | NA | Y | NR | NR | NR | Fair |
| Nguyen 2022 | Y | Y | NR | Y | N | NR | Y | NR | NA | NA | Y | NR | NR | NR | Fair |
| Ocon 2022 | Y | Y | NR | Y | N | NR | NR | NR | NA | NA | Y | NR | NR | NR | Poor |
| Ordaya 2022 | Y | Y | NR | Y | N | NR | Y | N | NA | NA | NR | NR | NR | NR | Poor |
| Ordaya 2023 | Y | Y | NR | Y | N | NR | NR | N | NA | NA | Y | NR | NA | NR | Poor |
| Totschnig 2022 | Y | Y | Y | Y | N | NR | Y | NR | NA | NA | Y | NR | Y | NR | Fair |
| Ocon 2023 | Y | Y | NR | Y | N | NR | Y | NR | NA | NA | Y | NR | NR | NR | Fair |
| Connolly 2023 | Y | Y | NR | Y | N | NR | Y | NR | NA | NA | Y | NR | NR | NR | Fair |
| Jurdi 2022 | Y | Y | NA | Y | N | Y | Y | NR | NA | NA | Y | NR | NR | NR | Fair |
| Alejo 2023 | Y | Y | NR | Y | N | NR | Y | NR | NA | NA | Y | NR | NR | NR | Fair |
| Benotmane 2022 | Y | Y | NR | Y | N | NR | Y | NR | NA | NA | Y | NR | NR | NR | Fair |
| Bertrand 2022 | Y | Y | NR | Y | N | NR | Y | NR | NA | NA | Y | NR | NR | NR | Fair |
| Chen 2022 | Y | Y | NA | Y | N | Y | Y | NR | NA | NA | Y | NR | NR | NR | Fair |
| Cochran 2023 | Y | Y | NR | Y | N | NR | Y | NR | NA | NA | NR | NR | NR | NR | Poor |
| Davis 2023 | Y | Y | NA | Y | N | Y | Y | NR | NA | NA | Y | NR | NR | NR | Fair |
| Gottlieb 2023 | Y | Y | NA | Y | N | Y | Y | NR | NA | NA | Y | NR | NR | NR | Fair |
| Kaminski 2022 | Y | Y | NA | Y | N | NR | Y | NR | NA | NA | Y | NR | NR | NR | Fair |
| Marchesi 2023 | Y | Y | NA | Y | N | NR | Y | NR | NA | NA | NR | NR | NR | NR | Poor |
| Young-Xu 2022 | Y | Y | NA | Y | N | NR | Y | NR | NA | NA | Y | NR | NR | NR | Fair |
| Demolder 2024 | Y | Y | N | N | N | NR | Y | NR | NA | Y | Y | NR | NR | NR | Poor |
| Fraczkiewicz 2024 | Y | Y | NR | Y | NR | NR | Y | N | NR | N | Y | NR | NR | NR | Fair |
| AA Roppelt 2023 | Y | Y | NR | Y | NR | NR | Y | N | NR | N | Y | NR | NR | NR | Fair |
| Lichvar 2023 | Y | Y | NR | NR | NR | NR | N | NR | NR | NR | NR | NR | NR | NR | Poor |
| Lombardi 2023 | Y | Y | Y | N | NR | NR | N | N | NR | N | Y | NR | Y | N | Fair |
| Benotmane 2023 | Y | Y | NR | Y | NR | NR | NR | N | NR | N | Y | NR | Y | N | Fair |
| Trepl 2024 | Y | Y | NR | Y | NR | NR | NR | NR | NR | N | Y | NR | Y | N | Fair |
| Angelico 2023 | Y | Y | Y | Y | NR | Y | Y | NR | NR | Y | Y | NR | Y | N | Fair |
| Sindu 2023 | Y | Y | Y | Y | NR | NR | NR | NR | NA | N | Y | NR | Y | N | Fair |

Abbreviations: Y, yes; N, no; NA, not applicable; NR, not reported,

(1) Objective clearly stated; (2) study population described; (3) participation rate; (4) adherence to inclusion and exclusion criteria; (5) sufficient sample size; (6) prior measure of outcomes; (7) sufficiency of timeframe; (8) examination different levels of the exposure; (9) exposure measures clearly defined; (10) exposure(s) assessed more than once; (11) outcome measures clearly defined; (12) outcome assessors blinded; (13) loss to follow-up; (14) confounding variables measured and adjusted

**Supplementary Table 3: Aim and summary of findings of the included studies**

| Study ID | Aim | Main findings |
| --- | --- | --- |
| Al-Obaidi 2023 | To evaluate the effectiveness of Tixagevimab−Cilgavimab in decreasing hospitalizations and mortality from COVID-19 among high-risk immuno-compromised hosts. | In immunocompromised patients with inadequate immune responses to vaccination, tixagevimab, and cilgavimab protect against COVID-19 sequelae. |
| Calabrese 2022 | To investigate the outcome of covid 19 infection in patients with immune-mediated inflammatory disease undergoing B cell depleting therapy or with inborn errors of humoral immunity who received tixagevimab/ cilgavimab as a pre-exposure prophylaxis. | The data showed that among 12 patients 11 had mild symptoms and only one patient had severe symptoms. |
| Jakimovski 2023 | Assessing the effectiveness of tixagevimab and cilgavimab in preventing COVID-19 infection in immunocompromised neuroinflammatory patients over 6 months | Tixagevimab and cilgavimab can be used as a prophylaxis against COVID-19 infection in neuroinflammatory patients with inadequate immunity who are treated with anti-CD20 monoclonal antibodies and sphingosine-1-phosphate modulators. |
| Jondreville 2022 | To assess the efficacy of tixagevimab/ cilgavimab as a prophylaxis against covid-19 infection in patients of allogeneic hematopoietic stem cell transplantation. | The results suggest the use of tixagevimab/ cilgavimab as a prophylaxis against covid-19 infection in patients of allogeneic hematopoietic stem cell transplantation who did not develop humoral immunity after getting vaccinated. |
| Karaba 2023 | To assess the efficacy of tixagevimab/ cilgavimab as a prophylaxis against COVID-19 infection in patients of solid transplant and neutralizing activity over 3 months. | The results support using tixagevimab/ cilgavimab as adjunct to vaccination to increase humoral immunity against Omicron BA. infection solid organ transplant recipients. |
| Kertes 2023 | Assessment of tixagevimab and cilgavimab efficacy to reduce hospitalization and mortality rates in immunocompromised patients during Omicron wave. | In immunocompromised, tixagevimab and cilgavimab may provide protection against Omicron variant infection and severe illness. |
| Montgomery 2022 | To assess safety and efficacy in mildly and moderately non hospitalized COVID-19 infected patients to prevent mortality | A single intramuscular dose of tixagevimab-cilgavimab gave statistically and clinically significant reduced mortality in comparison to placebo in mild to moderate COVID-19. Also, early intervention with tixagevimab-cilgavimab might lead to better results. |
| Nguyen 2022 | To assess the efficacy of tixagevimab-cilgavimab as a prophylaxis against COVID-19 infection in immunocompromised patients. | The results support the efficacy of tixagevimab-cilgavimab as a prophylaxis against COVID-19 infection in immun0compromised patients. |
| Ocon 2022 | To assess tixagevimab and Cilgavimab efficacy in patients with hematological malignancy. | Tixagevimab and Cilgavimab may be effective as adjuvant with vaccines and standard protocols. Hematological malignancy patients who received Tixagevimab and Cilgavimab showed a low rate of infection and hospitalization |
| Ordaya 2022 | To assess the characteristics of immunosuppressed patients who got COVID-19 infection after tixagevimab-cilgavimab as a prophylaxis. | Further investigations are needed to assess the effectiveness of tixagevimab-cilgavimab in immunocompromised patients. patients should complete the immunization doses and use makes as preventive actions. |
| Ordaya 2023 | Identifying the genotype and or predicted phenotype of COVID-19 subvariants in COVID-19 infected immunocompromised patients after receiving a prophylactic dose of tixagevimab-cilgavimab. | The infection with COVID-19 in immunocompromised patients was mainly due to COVID-19 Omicron subvariants with S codon substitutions associated with genotypic and/or predicted phenotypic which are less susceptible to tixagevimab-cilgavimab. |
| Totschnig 2022 | To assess the outcome of immunocompromised patients who received tixagevimab-cilgavimab and sotrovimab as a prophylaxis for COVID-19. | Tixagevimab-cilgavimab and sotrovimab succeeded to reduce incidence of COVID-19 breakthrough infections however more randomized trials are required to reassure the effectiveness |
| Ocon 2023 | To assess the efficacy of tixagevimab-cilgavimab in protecting against symptomatic COVID-19 infection in rheumatologic patients taking rituximab. | Tixagevimab and cilgavimab were effective in protecting rheumatologic patients taking rituximab against COVID-19 infection. |
| Connolly 2023 | To assess the safety of tixagevimab-cilgavimab in patients with inadequate immunity and their neutralization capability against omicron variant of concern. responses of binding antibody. | Tixagevimab-cilgavimab showed low neutralizing capacity towards some Omicron sub lineages as BA.4 and BA.5. the data confirm the necessity for the multi protective actions in immunocompromised patients. |
| Al Jurdi 2022 | To assess the safety and efficacy of tixagevimab-cilgavimab in lowering incidence of COVID-19 breakthrough infection in solid organ transplant patients. | Tixagevimab-cilgavimab is well tolerated and reduces the breakthrough infection incidence in solid organ transplant patients. |
| Alejo 2023 | To assess safety, efficacy and tolerability of tixagevimab-cilgavimab in solid organ transplant patients. | The results showed that tixagevimab and cilgavimab have good safety and tolerability in solid organ transplant patients. |
| Benotmane 2022 | Reporting COVID-19 breakthrough infections in kidney transplant recipients who got 150 mg of tixagevimab and 150 mg of cilgavimab as a prophylaxis. | Using 150 mg of tixagevimab and 150 mg of cilgavimab as a prophylaxis in kidney transplant recipients was not enough in protecting against COVID-19 breakthrough infection. Further studies are required to determine the optimal dose in order to achieve the optimal protective actions. |
| Bertrand 2022 | To evaluate the effectiveness of monoclonal antibody (tixagevimab-cilgavimab) as a prophylaxis against COVID-19 infection in kidney transplant recipients. | The data showed that the use of monoclonal antibody (tixagevimab-cilgavimab) in kidney transplant recipient is a successful protective strategy against COVID19 infection. |
| Chen 2022 | To determine the number of new cases who got a symptomatic infection and the number of hospitalizations in immunocompromised who received tixagevimab/cilgavimab as a prophylaxis during Omicron wave. | The effectiveness of tixagevimab-cilgavimab in the Omicron wave is still challenging because of the differing immunization coverage, several regimens, and different variants. |
| Cochran 2023 | To investigate COVID-19 outcomes in solid organ transplant patients taking tixagevimab-cilgavimab. | Solid organ transplants recipient who received tixagevimab-cilgavimab prophylaxis were less likely to get COVID-19 infection and hospitalization was rare. |
| Davis 2023 | To assess the effectiveness of tixagevimab-cilgavimab as a prophylaxis in protection against covid 19 infection in B-cell malignancies patients. | Malignancies patients are susceptible to breakthrough infections even after receiving tixagevimab-cilgavimab as prophylaxis however the mortality and hospitalization incidence was lower. It is recommended that these patients do multi preventative actions such as complete vaccination doses. Future investigations are required to assess the efficacy of repeated dosing strategy and if effectiveness is consistent against viral variants. |
| Gottlieb 2023 | To assess effectiveness of tixagevimab-cilgavimab as a prophylaxis to prevent COVID-19 infection in lung transplant recipients. | The group who received tixagevimab-cilgavimab showed less incidence of COVID-19 infection. In patients who were at higher risk due to old age and comorbidities, tixagevimab-cilgavimab showed a favorable effect on the outcomes of COVID-19 and reduced mortality in this group of patients. |
| Kaminski 2022 | To assess the effectiveness of tixagevimab-cilgavimab as a prophylaxis to reduce incidence of covid 19 break through number of hospitalizations and mortality in kidney transplant recipient during Omicron wave. | Tixagevimab–cilgavimab reduced COVID-19 break through, incidence of hospitalization and mortality in kidney transplant recipient in comparison to the group who didn’t recieve tixagevimab–cilgavimab. |
| Levin 2022 | To evaluate the safety and effectiveness of tixagevimab-cilgavimab as a prophylaxis against COVID-19 inpatients at high risk to worse outcomes, insufficient response to the COVID-19 vaccination or both. | Without any obvious safety issues single dose of tixagevimab-cilgavimab was effective in preventing COVID-19 in high risk patients. |
| Levin 2023 | To evaluate the effectiveness of tixagevimab and cilgavimab as a post-exposure prophylaxis against symptomatic COVID-19 infection. | Tixagevimab and cilgavimab single intramuscular dose was safe but did not show effectiveness in patients who had exposure index within 8 days. The data of the patients who were PCR–negative or with missing RT-PCR result at baseline showed favorable effect in preventing symptomatic COVID-19 infection. |
| Marchesi 2023 | Comparison of symptomatic COVID-19 infection outcomes in patients who received tixagevimab-cilgavimab as a prophylaxis with matched pairs who do not receive. | Tixagevimab-cilgavimab prophylaxis reduced mortality and hospitalization rates in hematological malignancy patients |
| Young-Xu 2022 | Evaluation of tixagevimab-cilgavimab as a prophylaxis against covid 19 and severe illness in immunocompromised patients. | Tixagevimab-cilgavimab succeeded to reduce the incidence of COVID-19 infection, mortality and hospitalization in immunocompromised patients during Omicron wave. |
| Lombardi 2023 | To assess clinical outcomes and time to nasal swap negativity in a cohohort of immunocompromised patients treated with Tixagevimab-cilgavimab. | Early treatment with Tixagevimab-cilgavimab in a small group of immunocompromised patients showed favourable outcomes, with no significant difference compared to similar patients treated with other mABs |
| Benotmane 2023 | To evaluate the use of Tixagevimab-cilgavimab as an early treatment for Covid-19 in kidney transplant recipient. | Administration of Tixagevimab-cilgavimab may be clinically useful in patients with Covid-19 and kidney transplant recipients. |
| Trepl 2024 | To compare the incidence of SARS-COV-2 infection between allo-HCT recipients who received Tixagevimab-cilgavimab and others who did not | Treatment with Tixagevimab-cilgavimab has potential efficacy in protecting from covid-19. |
| Angelico 2023 | To assess safety and efficacy of PreEP with Tixagevimab-cilgavimab in kidney and liver transplant recipient. | PreEP with Tixagevimab-cilgavimab might be effective in preventing covid-19 severe disease and hospitalization. |
| Sindu 2023 | To determine whether Tixagevimab-cilgavimab reduced the incidence and disease severity of SARS-COV-2 infection in lung transplant recipient during Omicron wave. | Tixagevimab-cilgavimab may reduce the incidence of covid-19 in lung transplant recipient, but it did not reduce disease severity during Omicron wave. |
| Demolder 2024 | To evaluate COVID-19 breakthrough infections and COVID-19 related complications after PrEP in LTx recipients who received PrEP between June 2022 and December 2022, when Omicron BA.5 was the dominant circulating SARS-CoV-2 variant. | 11.0% of lung transplant recipients developed breakthrough SARS-CoV-2 infection/COVID- 19 within the initial 3 months post-PrEP, which increased to 17.4% within 6 months. Notably, 27.6% of the patients with breakthrough infection within the first 3months required hospitalization, while this number increased to 52.9% for those with breakthrough infection during the subsequent 3 months of follow-up. |
| Fraczkiewicz 2024 | To explore the use of TIXA/CILGA as pre-exposure prophylaxis and treatment for COVID-19 among immunocompromised pediatric patients | TIXA/CILGA has shown to be successful in both COVID-19 prophylaxis and treatment in immunocompromised children, including those under 12 years of age. |
| AA Roppelt 2023 | To evaluate the efficacy and safety of pre-exposure prophylaxis of new SARS-CoV-2 infection in PIDs with the combination of tixagevimab/cilgavimab. | The use of tixagevimab/cilgavimab in patients with primary immunodeficiencies is effective as pre-exposure prophylaxis and reduces the risk of severe COVID-19. |
| Lichvar 2023 | To identify factors contributing to breakthrough COVID-19 infections in SOTR receiving T/C | The study found a low rate of breakthrough COVID-19 infections (7.7%) among SOTR who received T/C prophylaxis. |

**Tixagevimab-Cilgavimab (T/C)**: Monoclonal antibody combination used for COVID-19 prophylaxis; **COVID-19**: Coronavirus Disease 2019; **B-cell**: Type of white blood cell important for immune response; **RT-PCR**: Reverse Transcription Polymerase Chain Reaction; **Omicron BA.**: A variant of the SARS-CoV-2 virus; **mABs**: Monoclonal Antibodies; **PrEP**: Pre-exposure Prophylaxis; **PIDs**: Primary Immunodeficiencies; **SOTR**: Solid Organ Transplant Recipients; **allo-HCT**: Allogeneic Hematopoietic Cell Transplantation; **SARS-CoV-2**: Severe Acute Respiratory Syndrome Coronavirus 2.

**Supplementary Figure 1: Risk of bias assessment of the included studies using the Rob2 tool**


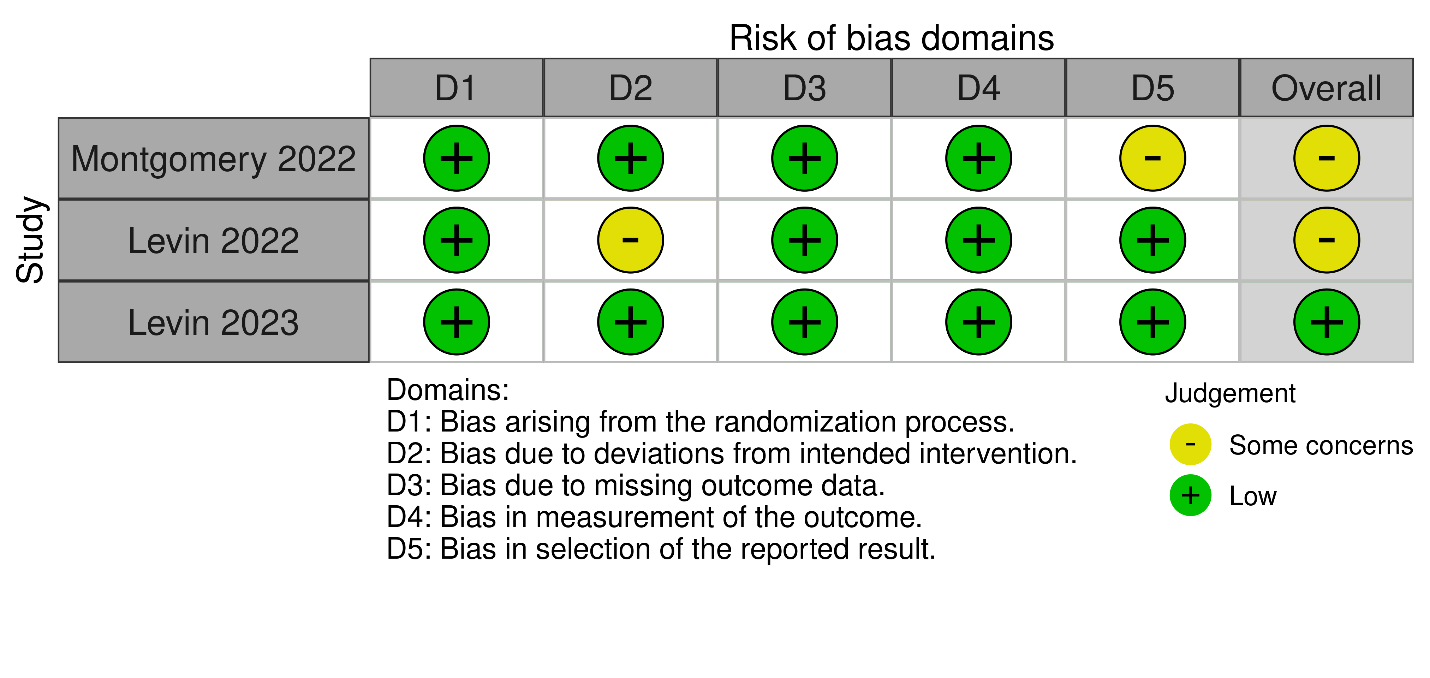


**Supplementary Figure 2: Leave-one-out analysis of the risk of infection in people taking tixagevimab-cilgavimab vs control**

**
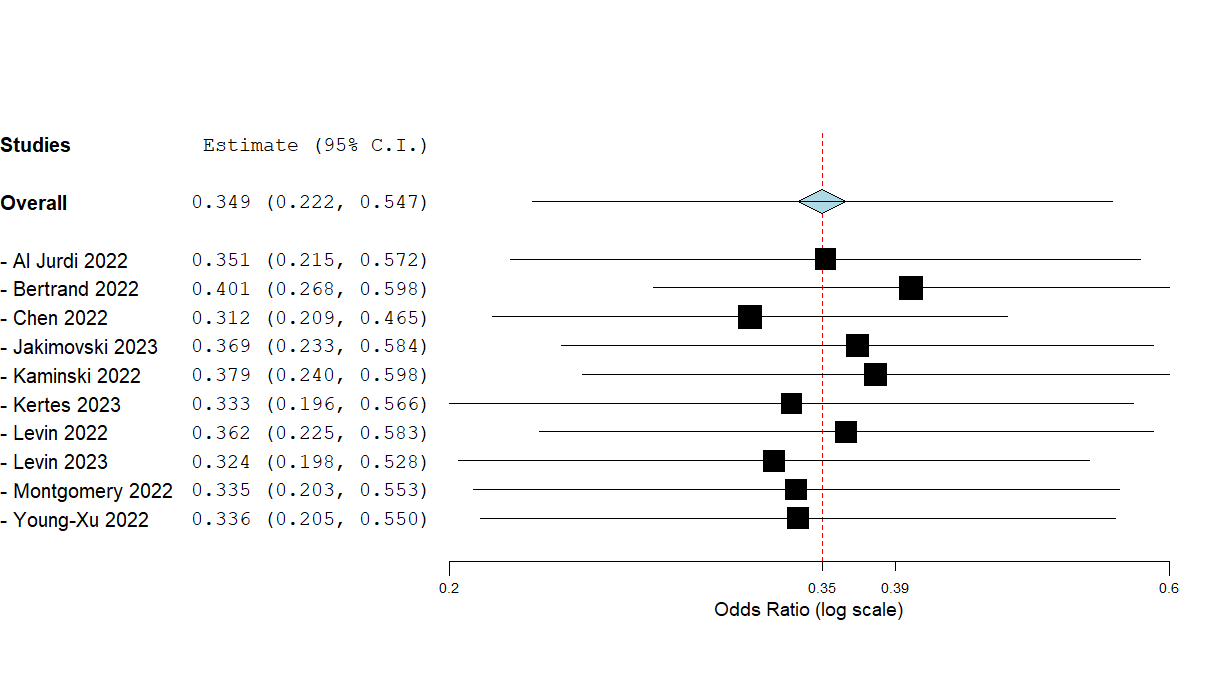
**

**Supplementary Figure 3: Leave-one-out analysis of COVID-19 specific hospitalization in people taking tixagevimab-cilgavimab vs control**

**
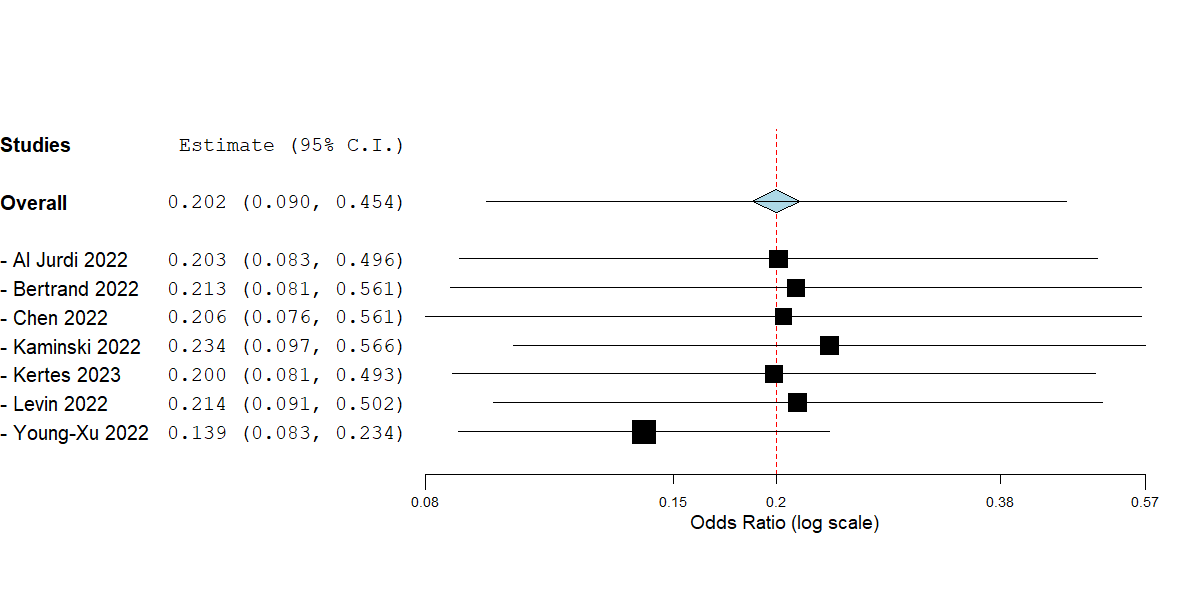
**

**Supplementary Figure 4: Comparison between tixagevimab/cilgavimab and placebo regarding the occurrence of (A) adverse events and (B) severe adverse events**

| 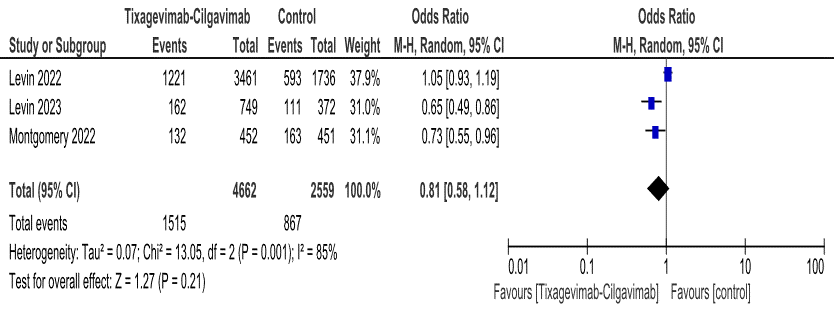 |
| --- |
|  |

**(b)**

**
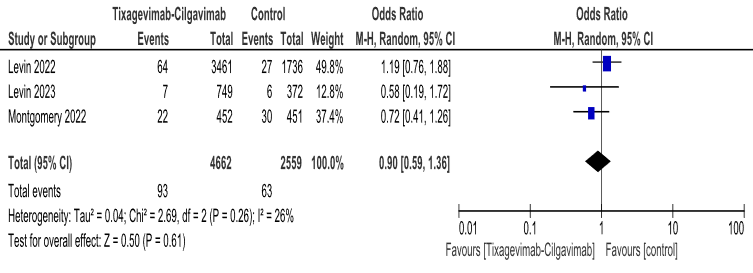
**

**Supplementary Figure 5: Publication bias using funnel plot for risk of SARS-CoV-2 infection outcome**

**
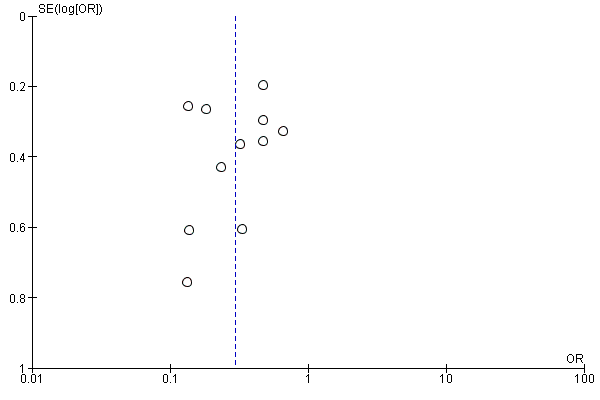
**
